# *KRAS*, *BRAF* and *PIK3CA* Mutations and the Loss of PTEN Expression in Chinese Patients with Colorectal Cancer

**DOI:** 10.1371/journal.pone.0036653

**Published:** 2012-05-07

**Authors:** Chen Mao, Junhua Zhou, Zuyao Yang, Yafang Huang, Xinyin Wu, Hong Shen, Jinling Tang, Qing Chen

**Affiliations:** 1 Department of Epidemiology, School of Public Health and Tropical Medicine, Southern Medical University, Guangzhou, Guangdong, People's Republic of China; 2 Division of Epidemiology, School of Public Health and Primary Care, the Chinese University of Hong Kong, Hong Kong, People's Republic of China; 3 Department of Pathology, Southern Medical University, Guangzhou, Guangdong, People's Republic of China; Baylor College of Medicine, United States of America

## Abstract

**Background:**

To investigate the frequency and relationship of the *KRAS*, *BRAF* and *PIK3CA* mutations and the loss of PTEN expression in Chinese patients with colorectal cancer (CRC).

**Methodology/Principal Findings:**

Genomic DNA was extracted from the formalin-fixed paraffin-embedded (FFPE) tissues of 69 patients with histologically confirmed CRC. Automated sequencing analysis was conducted to detect mutations in the *KRAS* (codons 12, 13, and 14), *BRAF* (codon 600) and *PIK3CA* (codons 542, 545 and 1047). PTEN protein expression was evaluated by immunohistochemistry on 3 mm FFPE tissue sections. Statistical analysis was carried out using SPSS 16.0 software. The frequency of *KRAS*, *BRAF* and *PIK3CA* mutations and loss of PTEN expression was 43.9% (25/57), 25.4% (15/59), 8.2% (5/61) and 47.8% (33/69), respectively. The most frequent mutation in *KRAS*, *BRAF* and *PIK3CA* was V14G (26.7% of all mutations), V600E (40.0% of all mutations) and V600L (40.0% of all mutations), and H1047L (80.0% of all mutations), respectivly. Six *KRAS* mutatant patients (24.0%) harbored *BRAF* mutations. *BRAF* and *PIK3CA* mutations were mutually exclusive. No significant correlation was observed between the four biomarkers and patients' characteristics.

**Conclusions/Significance:**

*BRAF* mutation rate is much higher in this study than in other studies, and overlap a lot with *KRAS* mutations. Besides, the specific types of *KRAS* and *PIK3CA* mutations in Chinese patients could be quite different from that of patients in other countries. Further studies are warranted to examine their impact on prognosis and response to targeted treatment.

## Introduction

Two monoclonal antibodies (MoAb) targeted at epidermal growth factor receptor (EGFR), the chimeric IgG1 MoAb cetuximab and the fully humanized IgG2 panitumumab, have proven to be effective in combination with chemotherapy or as single agent for treatment of metastatic colorectal cancer (mCRC) [1], [2], [3]. However, the efficacy of MoAb is not consistent for every patient; some patients experience dramatic response to MoAb, whereas others show no response [4], [5], [6]. In order to facilitate selection of mCRC patients who may benefit from anti-EGFR MoAbs treatments, there is a clear need for identifying predictive biomarkers that indicate likelihood of response amongst potential recipients.

It has been reported that oncogenic activations of intracellular signaling pathways downstream of EGFR, including the RAS-RAF-MAPK and PI3K-PTEN-AKT signaling pathways, are important mechanisms for generating resistance to anti-EGFR MoAbs. In the RAS-RAF-MAPK pathway, active mutations of *KRAS* or *BRAF* are not uncommon, as such mutations are present in 35.0–45.0% and in 4.0–15.0% of mCRC patients respectively [7]. In the PI3K-PTEN-AKT pathway, mutations of *PI3KCA* or loss of PTEN expression are observed in 10.0–18.0% and 19.0–42.0% of mCRC patients respectively [7]. Mutations of *PIK3CA*, may coexist with either *KRAS* or *BRAF* within the same tumor [8], but *KRAS* and *BRAF* mutations appear to be mutually exclusive [7].

To date, *KRAS* mutations have been identified as a predictive marker of resistance to anti-EGFR MoAbs in patients with mCRC, and the use of anti-EGFR MoAbs is now restricted to mCRC patients with wild-type *KRAS* [9]. However, the occurrence of *KRAS* mutations only accounts for approximately 30% to 40% of nonresponsive patients [10]. In patients with *KRAS* wild-type tumors, it remains unclear why a large number of patients are still not responsive to the treatment. More recently, other oncogenic mutations, such as *BRAF* [11], [12], *PIK3CA* mutations [10] or loss of PTEN expression [12], [13], are found likely to be promising predictors for the resistance in mCRC patients with wild-type *KRAS*.

Most of the studies that investigated the predictive value of *KRAS*, *BRAF*, *PIK3CA* mutations and loss of PTEN expression were performed in western countries. Little is known about the relation of these biomarkers with the clinical outcomes of MoAb treatment in Chinese patients with mCRC. We did not even know the frequency of these biomarkers occurred in Chinese patients. In this study, we investigated the status of *KRAS*, *BRAF*, *PI3KCA* mutation and PTEN expression in primary tumor from 69 Chinese mCRC patients, to clarify the rate of mutations and to detect the correlation between mutations and clinicopathological factors.

## Materials and Methods

### Patients and tissue samples

The analysis was conducted in 69 patients with histologically confirmed colorectal cancer (40 males and 29 females with a mean age of 54 years) who underwent tumor resection at Nanfang Hospital during the period of July 2010 to March 2011. Sixty-nine primary tumor samples were collected from surgical specimens. All of the collected samples are formalin-fixed paraffin-embedded (FFPE) tissues. This study was approved by the Institutional Ethics Committee of the Nanfang Hospital and was performed according to the institutional Guidelines. Written consent was given by the patients for their information to be stored in the hospital database and used for research. In our study, written informed consent was not obtained from the participants, because the study was retrospective and our data was analyzed anonymously. A summary of the demographic and clinicopathological data was listed in Table 1. Patients who ever smoked at least one cigarette per day for at least 6 months were categorized as smokers, including current smokers and previous smokers. The rest of patients were categorized as non-smokers. We considered patients who have at least 3 drinks per week on average in the past two years as drinkers, while the rest of patients were categorized as non-drinker.

**Table 1:** Characteristics of 69 patients with metastatic colorectal cancer.

Characteristics	
Sex-No. (%)	
Male	40(58.0)
Female	29 (42.0)
Missing	0(0.0)
Age	
≤65-No. (%)	57(82.6)
>65-No. (%)	12(17.4)
Missing-No. (%)	0(0)
Mean±SD-yr	54.0±12.0
Range-yr	31.0–78.0
Drinking History-No. (%)	
Yes	9(13.0)
No	58(84.1)
Missing	2(2.9)
Smoking History-No. (%)	
Yes	16 (23.2)
No	51(73.9)
Missing	2(2.9)
Primary Tumor Site -No. (%)	
Right colon	14(20.3)
Left colon	20(29.0)
Rectum	30(43.5)
Missing	5(7.2)
Tumor type-No. (%)	
Mucinous	11(15.9)
Non-mucinous	56(81.2)
Missing	2(2.9)
Tumor Differentiation-No. (%)	
G1	13(18.8)
G2	10(14.5)
G3	29(42.0)
G4	3(4.3)
Missing	14(20.3)
T-No. (%)↑	
T2	5(7.2)
T3	44(63.8)
T4	17(24.6)
Missing	3(4.3)
N-No. (%)↑	
N0	36(52.2)
N1	15(21.7)
N2	15(21.7)
Missing	3(4.3)

Legend.

↑ Sixth edition of the AJCC/UICC TNM staging systems was applied.

### DNA extraction and mutational analysis of KRAS, BRAF and PIK3CA

Two appropriate FFPE samples were selected from each patient. For every sample, three 5–10 µm sections were prepared. Genomic DNA was extracted by a standard SDS-proteinase K procedure. After extraction, DNA was purified.

We searched for mutations in *KRAS* exon 2, *BRAF* exon 15 and *PIK3CA* exons 9 and 20. *KRAS* exon 2 includes codons 12, 13 and 14, *BRAF* exon 15 includes codon 600, *PIK3CA* exon 9 includes codons 542 and 545 and *PIK3CA* exon 20 includes codon 1047, where the large majority of mutations occur in these genes [11]. Ten types of mutations in *KRAS* codons 12, 13 and 14 (G12C, G12D, G12V, G12R, G12A, G12G, G13D, G13G, V14G and V14A), 4 types of mutations in *BRAF* codon 600 (V600E, V600Q, V600L and V600V), 4 types of mutations in *PIK3CA* codons 542 and 545 (E542K, E545K, E545G and E545A) and two types of *PIK3CA* codon 1047 (H1047R and H1047L) were detected. The nucleotide sequence corresponding to every exon was amplified from extracted genomic DNA. Table 2 shows the list of primers used for each exon. Conditions for the amplification of exon-specific regions from genomic DNA by PCR have been described in previous study [11]. PCR products were subjected to automated sequencing by ABI PRISM 3730 (Applied Biosystems, Foster City, CA, USA). All mutated cases were confirmed twice with independent PCR reactions. New data was not generated in our study. The results for mutation analyses are given in appendix S1 and S2 (Figures for sequencing results).

**Table 2:** The Primers used in PCR amplification and sequencing.

KRAS-Exon2-Forward	GGTGGAGTATTTGATAGTGTATTAACC
KRAS-Exon2-Reverse	AGAATGGTCCTGCACCAGTAA
BRAF-Exon15-Forward	TGCTTGCTCTGATAGGAAAATG
BRAF-Exon15-Reverse	AGCATCTCAGGGCCAAAAAT
PIK3CA-Exon9-Forward	GGGAAAAATATGACAAAGAAAGC
PIK3CA-Exon9-Reverse	CTGAGATCAGCCAAATTCAGTT
PIK3CA-Exon20-Forward	CTCAATGATGCTTGGCTCTG
PIK3CA-Exon20-Reverse	TGGAATCCAGAGTGAGCTTTC

### PTEN expression

PTEN protein expression was evaluated by immunohistochemistry on 3 *mm* FFPE tissue sections as reported in previous study [13]. The monoclonal anti-mouse anti-human PTEN antibody was applied at 1: 50 dilution. Each run included appropriate positive and negative control slides. A semi-quantitative score was given to PTEN staining of tumor tissue by two independent pathologists without knowledge of clinical data or results of molecular analyses: negative(−), no staining at all; weak(+), weak staining regardless of positive cell percentages or moderate staining of ≤30% of cells; moderate (++), moderate staining of >30% of cells or strong staining of ≤50% of cells; strong (+++), strong staining of >50% of cells. Tumors with PTEN scores of − , + or ++ were considered to have PTEN loss. The figures for immunohistochemical analysis are given in appendix S3.

### Statistical analysis

Statistical analysis was conducted using SPSS 16.0 (SPSS, Inc., Chicago, IL). The χ^2^ test and Fisher's exact test were used to compare the proportion of *KRAS*, *BRAF PIK3CA* mutations and loss of PTEN expression among different clinicopathologic groups. To investigate the effects of covariates on gene mutations, multiple logistic regression analysis using a forward stepwise (likelihood ratio) method was done with odds ratio (OR) calculated. Initial testing included age, gender, smoking history, drinking history, tumor site and differentiation. Only variables showing statistically significant association with gene mutations were subjected to final regression analysis. The two-sided significance level was set at P<0.05.

## Results

### *KRAS* mutation

*KRAS* mutational status was tested in 57 tumor tissues, of which 25 (43.9%) harbored at least one mutation at codons 12, 13 or 14. The spectrum of these mutations was summarized in Table 3. Eighteen (31.6%) tissues had a mutation at codon 12, 4 (7.0%) at codon 13 and 8 (14.0%) at codon 14. The most frequent mutation was V14G, which represented 26.7% of all mutations, followed by G12D (20.0% of all mutations). Five tissues had concomitant mutations at two codons (Appendix 1). We did not find any significant association between KRAS mutations and patients' characteristics by univariate analysis (Table 4) and multivariate analysis (data not shown).

**Table 3:** The frequency of KRAS, BRAF and PIK3CA mutations according to different patterns.

Patterns of mutations	No. of patients (%)
KRAS Exon2 (codon 12)	
G12C	1(1.8)
G12D	6(10.5)
G12V	4(7.0)
G12R	1(1.8)
G12A	3(5.3)
G12G	3(5.3)
Wild-type	39(68.4)
KRAS Exon2 (codon 13)	
G13D	1(1.8)
G13G	3(5.3)
Wild-type	53(93.0)
KRAS Exon2 (codon 14)	
V14G	8(14.0)
V14A	0(0.0)
Wild-type	49(86.0)
BRAF Exon15 (codon 600)	
V600E	6(10.2)
V600Q	2(3.4)
V600L	6(10.2)
V600V	1(1.70)
Wild-type	44(74.6)
PIK3CA Exon9 (codons 542 and 545)	
E542K	0(0.0)
E545K	0(0.0)
E545G	1(1.7)
E545A	0(0.0)
Wild-type	57(98.3)
PIK3CA Exon20 (codon 1047)	
H1047R	0(0.0)
H1047L	4(7.0)
Wild-type	53(93.0)

**Table 4:** Association of KRAS, BRAF and PIK3CA mutations and loss of PTEN expression with clinical and pathologic characteristics.

Variables	KRAS		BRAF		PIK3CA		PTEN expression	
	Mutations/Total number (%)	P	Mutations/Total number (%)	P	Mutations/Total number (%)	P	Loss/Total number (%)	P
Sex								
Male	13/33(39.4)	0.426	11/34(32.4)	0.154	3/35(8.6)	1.000	17/40(42.5)	0.298
Female	12/24(50.0)		4/25(16.0)		2/26(7.7)		16/29(55.2)	
Age								
<65	22/46(47.8)	0.370	12/48(25.0)	1.000	4/49(8.2)	1.000	28/57(49.1)	0.638
≧65	3/11(27.3)		3/11(27.3)		1/12(8.3)		5/12(41.7)	
Drinking History								
Yes	5/8(62.5)	0.436	3/9(33.3)	0.807	0/9(0.0)	1.000	3/9(33.3)	0.504
No	19/47(40.4)		11/48(22.9)		5/58(8.6)		30/58(51.7)	
Smoking History								
Yes	8/15(53.3)	0.375	4/16(25.0)	1.000	1/16(6.3)	1.000	6/16(37.5)	0.281
No	16/40(40.0)		10/41(24.4)		4/51(7.8)		27/51(52.9)	
Primary Tumor Site								
Right colon	5/11(45.5)	0.746	2/11(18.2)	0.701	1/14(7.1)	0.807	7/14(50.0)	0.321
Left colon	5/14(35.7)		5/16(31.3)		1/20(5.0)		7/20(35.0)	
Rectum	13/27(48.1)		6/27(22.2)		3/30(10.0)		17/30(56.7)	
Mucinous								
Yes	6/10(60.0)	0.423	2/9(22.2)	1.000	1/11(9.1)	1.000	6/11(54.5)	0.701
No	18/45(40.0)		12/48(25.0)		4/56(7.1)		27/56(48.2)	
Tumor Differentiation								
G1	4/11(36.4)	0.990	2/12(16.7)	0.631	2/13(15.4)	0.514	5/13(38.5)	0.725
G2	3/7(42.9)		3/7(42.9)		0/10(0.0)		5/10(50.0)	
G3	9/23(39.1)		6/25(24.0)		2/29(6.9)		16/29(55.2)	
G4	1/3(33.3)		1/3(33.3)		0/3(0.0)		1/3(33.3)	
T Stages								
T2	2/5(40.0)	0.883	1/5(20.0)	0.886	0/5(0.0)	0.680	3/5 (60.0)	0.879
T3	17/37(45.9)		10/37(27.0)		4/38(10.5)		22/44(50.0)	
T4	5/13(38.5)		3/14(21.4)		1/16(6.3)		8/17(47.1)	
N Stages								
N0	14/30(46.7)	0.553	10/30(33.3)	0.290	2/32(6.3)	0.143	19/36(52.8)	0.885
N1	6/12(50.0)		2/11(18.2)		0/12(0.0)		7/15(46.7)	
N2	4/13(30.8)		2/15(13.3)		3/15(20.0)		7/15(46.7)	

### *BRAF* mutation

**Figure 1:**
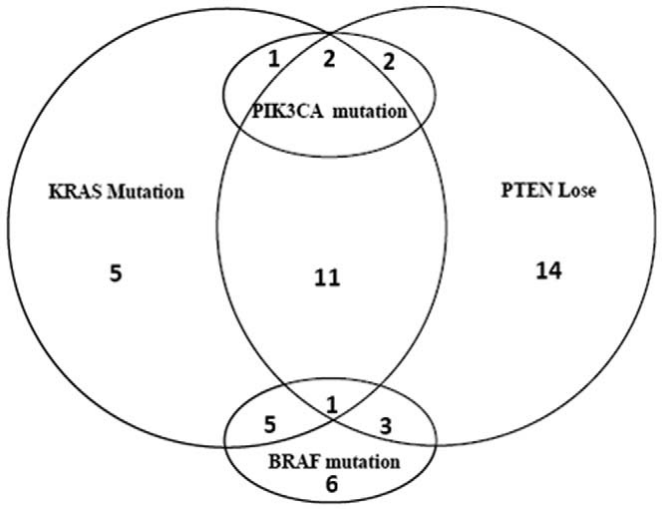
The interrelationship between four biomarkers.

We detected *BRAF* codon 600 mutations in 15 (25.4%) out of 59 tumor tissues. The most frequent mutation was V600E (40.0% of all mutations) and V600L (40.0% of all mutations) (Table 3). *BRAF* and *KRAS* mutations were not mutually exclusive, with 24.0% *KRAS* mutated patients and 29.0% wild-type *KRAS* patients harboring *BRAF* mutations (Figure 1). No significant association between *KRAS* mutations and patients' characteristics was found by univariate analysis (Table 4) and multivariate analysis (data not shown).

### *PIK3CA* mutation

The status of *PIK3CA* mutations was analyzed in 61 tumor tissues with 5 positive results, giving a total mutation rate of 8.2%. *PIK3CA* Exon9 mutation was seen in only one (1.7%) out of 58 tumor tissues tested (Table 3). By contrast, *PIK3CA* Exon20 mutations were identified in 4 out of 57 tumor tissues (7.0%), all being H1047L (Table 3). *KRAS* and *PIK3CA* mutations were not mutually exclusive (Figure 1). Three (12.0%) *KRAS* mutated patients had *PIK3CA* mutations, all located in Exon20, whereas two (6.3%) *KRAS* wild-type patients harbored *PIK3CA* mutations, one in Exon9 and the other in Exon20. Of note, *BRAF* and *PIK3CA* mutations were mutually exclusive in the present group of patients. No significant association between *PIK3CA* mutations and patients' characteristics was found by univariate analysis (Table 4) and multivariate analysis (data not shown).

### Loss of PTEN expression

We tested the PTEN expression in 69 tumor tissues. Loss of PTEN expression was detected in 33 of them (47.8%), and was not mutually exclusive with *KRAS*, *BRAF* or *PIK3CA* mutations (Figure 1). Fourteen (56.0%) *KRAS* mutated patients had loss of PTEN expression (Figure 1). We did not detect any statistically significant association between PTEN expression and patients' characteristics by univariate analysis (Table 4) and multivariate analysis (data not shown).

## Discussion

In this study, we detected various mutations of the KRAS, BRAF and PIK3CA genes as well as the loss of PTEN expression in 69 Chinese CRC patients. In addition, we also tried to correlate the mutations with some clinical and pathological features. Some previous studies have investigated the relationship between these molecular events and various clinicopathological characteristics. The results were however inconsistent. For example, Sartore-Bianchi et al found that *KRAS* mutations were significantly more in women than in men, while *PIK3CA* mutations and loss of PTEN were not significantly associated with sex, age or site of tumor [14]. In contrast, Barault et al and Benvenuti et al found that *PIK3CA* and *BRAF* mutations, but not mutations of *KRAS*, occur at a higher frequency in women than men [10]. In Chinese CRC patients, Shen et al found that gender was the only factor that showed an obvious relationship with *KRAS* mutations (female 44.7% vs male 28.2%, P = 0.037) [15]; Liou et al reported more frequent *KRAS* mutations in females and in non-smokers, and *KRAS* and *BRAF* mutations were significantly associated with the proximal location of cancer [16]. However, in the study of Li et al, *BRAF* mutation did not correlate with age, gender, histological type or Dukes' staging, but co-existent *KRAS* and *PIK3CA* mutations were more likely to develop into liver metastasis [17].

In the present study, we did not find any significant correlations between these molecular events and various clinicopathological features (Table 4), which may be partly attributable to the relatively small sample size. We observed some potential tendencies. For example, *KRAS* mutations and loss of PTEN seemed to be higher in female than in male patients. In addition, *KRAS*, *BRAF* and *PIK3CA* mutations appeared to be more frequent in those with a drinking or smoking history. However, larger studies are needed to draw a firm conclusion on these issues.

*KRAS* gene encodes a 21 kDa RAS protein, which is a member of the GTPases family involved in signal transduction processes. Mutations in the *KRAS* can constitutively activate the protein in signaling by eliminating the GTPase activity [15]. It has been established that *KRAS* mutations are predictive biomarker for the resistance to anti-EGFR monoclonal antibodies (MoAbs) treatment in terms of response rate, progression-free survival and overall survival. According to previous reports, the *KRAS* mutation rate of CRC patients varies from 20.0% to 50.0%, mostly about 35.0%–45.0% [10], [15]. In this study, 43.9% had a mutant *KRAS* genotype, which means that, if *KRAS* mutational status testing is applied to select candidates for anti-EGFR MoAbs treatment, the proportion of Chinese CRC patients that would be excluded is similar to that of other countries.

However, it should be noted that in this study, 14.0% of the patients had codon 14 mutations (V14G). Among the few studies that have taken interest in codon 14, a large series from US showed that codon 14 mutations (V14I) occurred in only 0.1% of the CRC patients [18]. And it is unknown whether these variants are of specific pathogenicity [18]. Thus, it would be interesting to see whether our results are reproducible in future Chinese patients with mCRC. More importantly, further studies are warranted to investigate the impact of codon 14 mutations on patients' prognosis and response to anti-EGFR MoAbs. If these mutations do not confer resistance to the treatment, then more Chinese mCRC patients may benefit from anti-EGFR MoAbs.

In previous reports from western populations, G12D transitions were the most frequently found type of *KRAS* codon 12 mutations, followed by G12V, G12C, G12S and G12A [18], [19]. However, in our study, the corresponding order is G12D, G12V, G12A, G12G and G12C, among which G12G was rarely seen in other studies. As for codon 13 mutations, the majority of them were G13D, followed by G13C and G13R in western populations [18], [19]. In the present study, only G13G, a newly found variant, and G13D were detected. These data suggests that there may be racial difference in the patterns of *KRAS* mutations. It has been reported that the use of cetuximab was associated with longer overall and progression-free survival among patients with chemotherapy-refractory colorectal cancer with G13D-mutated tumors than patients with other *KRAS*-mutated tumors [20]. Whether some of the rarely seen or new mutations found in our study are also associated with better treatment outcome remains unknown and deserves further investigation.

Similar to KRAS gene, *BRAF* also encodes proteins that act in the RAS-RAF-MAPK signaling pathway. Previous studies, both Western and Chinese, reported that *BRAF* mutations were detected in 5.0%–10.0% of CRC patients. Surprisingly, our study demonstrated a quite high mutation rate, 25.4%, for *BRAF*. A possible explanation is that most studies of *BRAF* mutation were focused on V600E only [21], whereas our study analyzed four types of mutations, i.e. V600E, V600Q, V600L and V600V. However, even in De Roock's study that detected D594G, V600E, V600M and K601E, the mutation rate was only 10.9% [22]. Therefore, the high mutation rate in our study may be due to other reasons, such as racial difference and environmental factors. There is yet no consensus on the predictive role of *BRAF* mutations in the anti-EGFR MoAbs treatment of mCRC. Some found that V600E mutation was associated with worse outcome in metastatic CRC patients treated with anti-EGFR MoAbs [23]. Others suggested that this mutation was just a general prognostic factor rather than a predictive factor specific to anti-EGFR monoclonal antibodies, because its relationship with poor prognosis is independent from the given treatment [24]. Mutations of *KRAS* and *BRAF* genes are frequently found to be mutually exclusive in colorectal cancer, both in Western and Chinese patients [25], [26]. Thus, in general, with a *KRAS* mutation rate of 40.0% and a *BRAF* mutation rate of 10.0%, one sixth or 16.7% of the *KRAS* wild-type patients harbored *BRAF* mutations. Of note, *BRAF* mutations overlap a lot with *KRAS* mutations in this study, with 29.0% of wild-type *KRAS* patients harboring *BRAF* mutations. If *BRAF* mutations were used to further select wild-type *KRAS* patients for anti-EGFR MoAbs treatment, then significantly more Chinese mCRC patients can be excluded.

The PIK3CA gene encodes the p110 catalytic subunit of PI3K that regulates the pathways [27]. In agreement with previous studies, we found that the mutation rate for *PIK3CA* is 8.7%, and *PIK3CA* mutations are coexistent with *KRAS* mutations [10], [23], [28]. Besides, we observed more mutations at exon 20 than at exon 9, which is consistent with studies of Chinese patients [29], [30] but quite different from the results from Western populations. This is very important, because exon 9 and exon 20 mutations differ greatly in affecting the response to anti-EGFR MoAbs. Our previous systematic review found that *PIK3CA* exon 20 mutations was associated with a lower response rate, shorter progression-free survival and overall survival and thus may be a potential biomarker for resistance to anti-EGFR MoAbs in *KRAS* wild-type mCRC, whereas *PIK3CA* exon 9 mutations seemed to have no such role [31]. Therefore, by testing *PIK3CA* mutation status, more Chinese mCRC patients can be prevented from receiving anti-EGFR MoAbs to which they are resistant.

In a retrospective consortium analysis of more than 1000 tumors gathered from seven European countries, De Roock found that the E542K, E545K and Q546K mutations at exon 9 accounted for 15.6%, 26.8% and 4.2% of all the *PIK3CA* mutations, while the H1047R and H1047L mutations at exon 20 accounted for 20.5% and 3.8% of all the mutations [22]. In the present study, however, the most frequent type of mutations we detected is H1047L, not the abovementioned hotspots, such as E542K, E545K or H1047R. This indicates that there may be large variations across different races. Interestingly, we found that every patient that undertaken *PIK3CA* mutation analysis harbored E542K and E545K mutations. We deemed these as “false positive” results, which has been reported by others [32].

The loss of PTEN expression, which was reported to occur in 19.0%–42.0% of Western and 30.0%–64.0% of Chinese CRC tumors [13], [33], [34], [35], [36], [37], induces an increase in PIP-3 concentration and *PIK3CA* pathway activation [23]. We detected the loss of PTEN expression in 47.8% of the patients, consistent with previous studies. Loss of PTEN expression has been reported to confer tumor resistance to anti-EGFR MoAbs [38]. However, since this PTEN loss can coexist with *PIK3CA* mutations, as shown by the present and other studies, it is often difficult to differentiate the contribution of loss of PTEN from that of *PIK3CA* mutations to the lack of response [28].

The strength of this study is the comprehensive analysis of four biomarkers in Chinese mCRC patients. However, the samples size is relatively small, rendering some of our findings inconclusive. Furthermore, we did not collect the data on treatment and clinical outcomes, which will be addressed in our future studies.

In summary, this study adds to the evidence that *KRAS* and *PIK3CA* mutations and the loss of PTEN expression in Chinese mCRC patients occur at a comparable level to that of Western patients. However, *BRAF* mutation rate is much higher in this study than in previous studies. In addition, the specific types of *KRAS* and *PIK3CA* mutations in Chinese population could be quite different from that of patients in other countries, especially considering the relatively high frequency of *KRAS* codon 14 mutations and *PIK3CA* exon 20 mutations. These findings have important implications for the personalized treatment of Chinese mCRC patients. Further studies are warranted to examine the impact of some types of *KRAS*, *BRAF* and *PIK3CA* mutations on prognosis and response to targeted treatment.

## Supporting Information

Appendix S1 **Patterns of KRAS, BRAF and PIK3CA mutations.**
(DOC)

Appendix S2 **Sequencing results for KRAS, BRAF and PIK3CA mutations.**
(DOCX)

Appendix S3 **Immunohistochemical staining of PTEN in colorectal cancer tissue and normal tissue.**
(DOC)
