# Supplementary material for: KRAS, BRAF and PIK3CA Mutations and the Loss of PTEN Expression in Chinese Patients with Colorectal Cancer
Source: PLoS One. 2012 May 7;7(5):e36653. doi: 10.1371/journal.pone.0036653 (PMC3346734; doi:10.1371/journal.pone.0036653)
Supplement: Appendix S1 — Patterns of KRAS, BRAF and PIK3CA mutations. (DOC) [file pone.0036653.s001.doc]

Appendix S1. Patterns of KRAS, BRAF and PIK3CA mutations

| Age | Sex | Location | Differentiation | KRAS mutations | | | BRAF mutations | PIK3CA mutations | | |
| --- | --- | --- | --- | --- | --- | --- | --- | --- | --- | --- |
|  |  |  |  | Codon 12 | Codon 13 | Codon14 | Codon 600 | Codon 542 | Codon 545 | Codon 1047 |
| 78 | M | R | H-M | Neg | Neg | V14G | V600E | Neg | Neg | Neg |
| 53 | M | C | M | Miss | Miss | Miss | Miss | Miss | Miss | Miss |
| 52 | F | C | M | Miss | Miss | Miss | Miss | Miss | Miss | Miss |
| 49 | M | C | H-M | Neg | Neg | Neg | V600E | Neg | Neg | Miss |
| 47 | F | C | H-M | Miss | Miss | Miss | Miss | Neg | Neg | Neg |
| 54 | M | C | H | Miss | Miss | Miss | Miss | Miss | Miss | Miss |
| 62 | M | C | M | Miss | Miss | Miss | Miss | Miss | Miss | Miss |
| 55 | M | R | M | Miss | Miss | Miss | Miss | Miss | Miss | Miss |
| 41 | F | R | M | G12G | Neg | Neg | V600E | Neg | Neg | Miss |
| 48 | F | C | M | G12R | Neg | V14G | V600Q | Neg | Neg | Neg |
| 65 | F | R | M | G12V | Neg | Neg | Neg | Neg | Neg | Neg |
| 57 | F | C | H-M | Miss | Miss | Miss | Miss | Miss | Miss | Miss |
| 73 | F | R | H-M | Miss | Miss | Miss | Miss | Neg | Neg | Neg |
| 67 | M | C | H-M | Neg | Neg | Neg | V600E | Neg | Neg | Neg |
| 42 | M | R | H-M | G12G | Neg | Neg | Neg | Neg | Neg | Neg |
| 49 | F | R | M | Neg | Neg | Neg | Neg | Neg | Neg | Neg |
| 65 | M | R | M | G12A | Neg | Neg | Neg | Neg | Neg | Neg |
| 38 | F | R | M | G12D | Neg | V14G | Neg | Neg | Neg | H1047L |
| 48 | M | R | H | G12A | Neg | Neg | Neg | Neg | Neg | H1047L |
| 59 | F | R | M | Neg | Neg | Neg | Neg | Neg | Neg | Neg |
| 38 | F | C | L | G12D | Neg | Neg | Neg | Neg | Neg | Neg |
| 38 | F | C | M | G12D | Neg | Neg | Neg | Neg | Neg | Neg |
| 49 | F | R | M | Neg | G13G | V14G | Neg | Neg | Neg | Neg |
| 50 | M | R | H | Neg | Neg | V14G | Neg | Neg | Neg | Neg |
| 33 | M | C | M | Neg | Neg | Neg | Neg | Neg | Neg | Neg |
| 36 | F | R | M | Neg | Neg | Neg | Neg | Neg | Neg | Neg |
| 75 | M | C | M | Neg | Neg | Neg | Neg | Neg | Neg | Neg |
| 43 | F | C | M | Neg | Neg | Neg | V600E | Neg | Neg | Neg |
| 60 | F | C | H-M | G12D | Neg | Neg | Neg | Neg | Neg | Neg |
| 57 | M | R | H | Neg | Neg | V14G | V600V | Neg | Neg | Neg |
| 64 | F | C | H | G12A | Neg | Neg | Neg | Neg | Neg | Neg |
| 54 | M | R | H | Neg | Neg | Neg | Neg | Neg | Neg | Neg |
| 61 | M | R | Miss | G12V | Neg | Neg | V600L | Neg | Neg | Neg |
| 38 | M | C | L | Neg | Neg | Neg | V600L | Neg | Neg | Neg |
| 60 | F | C | M | Neg | Neg | Neg | Neg | Neg | Neg | Neg |
| 49 | M | R | M | Neg | Neg | Neg | Neg | Neg | Neg | Neg |
| 50 | F | R | M | Neg | Neg | Neg | Neg | Neg | Neg | Neg |
| 51 | F | C | H | Neg | Neg | Neg | Neg | Neg | Neg | Neg |
| 32 | M | C | M | G12D | Neg | Neg | Neg | Neg | Neg | Neg |
| 39 | M | R | L | Neg | Neg | Neg | Neg | Neg | Neg | Neg |
| 64 | M | C | H | Neg | Neg | Neg | Neg | Neg | E545G | Neg |
| 42 | M | C | M | Neg | Neg | Neg | Neg | Neg | Neg | Neg |
| 31 | M | C | M | Miss | Miss | Miss | Miss | Miss | Miss | Miss |
| 68 | M | C | M | G12C | Neg | Neg | Neg | Neg | Neg | Neg |
| 47 | M | R | M | Neg | G13G | V14G | Neg | Neg | Neg | Neg |
| 58 | F | C | L | Neg | Neg | V14G | Neg | Neg | Neg | Neg |
| 72 | F | C | Miss | Neg | Neg | Neg | Neg | Neg | Neg | Neg |
| 47 | M | R | H-M | Neg | Neg | Neg | Neg | Neg | Neg | Neg |
| 72 | M | C | H | Neg | Neg | Neg | Neg | Neg | Neg | Neg |
| 64 | F | C | M | Neg | Neg | Neg | Neg | Neg | Neg | Neg |
| 58 | M | C | H | Neg | Neg | Neg | Neg | Neg | Neg | Neg |
| 67 | M | R | M | Neg | Neg | Neg | Neg | Neg | Neg | H1047L |
| 58 | F | C | M | Neg | Neg | Neg | V600L | Neg | Neg | Neg |
| 35 | M | R | Miss | Neg | Neg | Neg | Miss | Neg | Neg | Miss |
| 55 | F | C | M | Neg | G13D | Neg | Neg | Neg | Neg | H1047L |
| 64 | F | R | H-M | Neg | Neg | Neg | Neg | Neg | Neg | Neg |
| 67 | M | C | H | Neg | Neg | Neg | Neg | Neg | Neg | Neg |
| 65 | F | C | H | Miss | Miss | Miss | Neg | Miss | Miss | Miss |
| 56 | M | C | M | Miss | Miss | Miss | Neg | Miss | Miss | Neg |
| 39 | F | C | M | G12D | Neg | Neg | Neg | Neg | Neg | Neg |
| 54 | M | R | M | Miss | Miss | Miss | Neg | Neg | Neg | Miss |
| 35 | M | Miss | Miss | Neg | Neg | Neg | V600L | Neg | Neg | Neg |
| 67 | M | R | M | G12V | Neg | Neg | Neg | Miss | Miss | Neg |
| 61 | M | R | M | Neg | Neg | Neg | V600L | Neg | Neg | Neg |
| 57 | M | Miss | Miss | G12V | Neg | Neg | V600L | Miss | Miss | Neg |
| 78 | F | R | M | Neg | Neg | Neg | Neg | Neg | Neg | Neg |
| 69 | M | C | M | Neg | Neg | Neg | V600Q | Neg | Neg | Neg |
| 48 | M | R | M | Neg | Neg | Neg | V600E | Neg | Neg | Neg |
| 47 | M | R | M | G12G | G13G | Neg | Neg | Neg | Neg | Neg |

H-M, High-moderately; L, Low; M, Moderately; Neg, negative.
