# Supplementary material for: KRAS, BRAF and PIK3CA Mutations and the Loss of PTEN Expression in Chinese Patients with Colorectal Cancer
Source: PLoS One. 2012 May 7;7(5):e36653. doi: 10.1371/journal.pone.0036653 (PMC3346734; doi:10.1371/journal.pone.0036653)
Supplement: Appendix S2 — Sequencing results for KRAS, BRAF and PIK3CA mutations. (DOCX) [file pone.0036653.s002.docx]

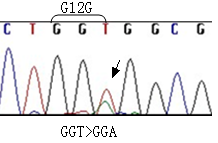

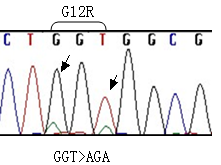

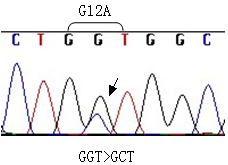


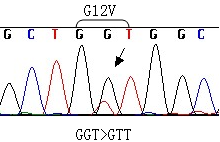

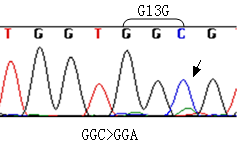

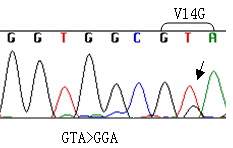

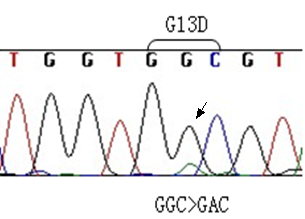

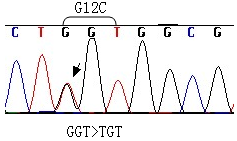

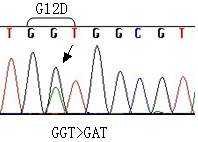


Figure 1. Sequencing results for *KRAS* mutations


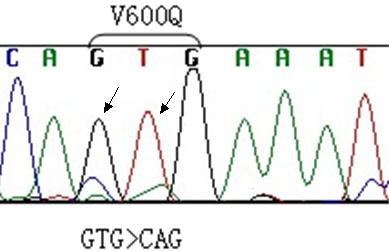

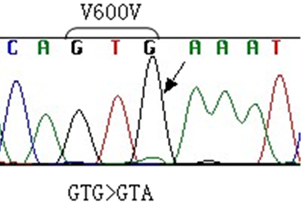

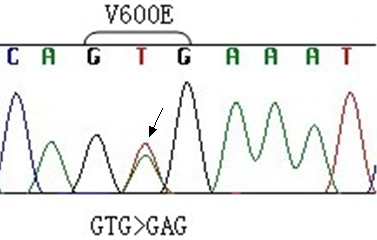

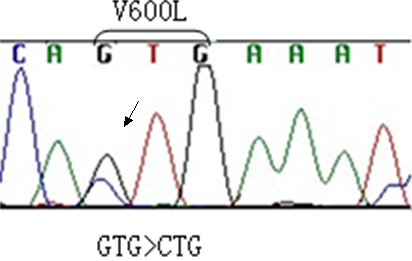


Figure 2. Sequencing results for *BRAF* mutations


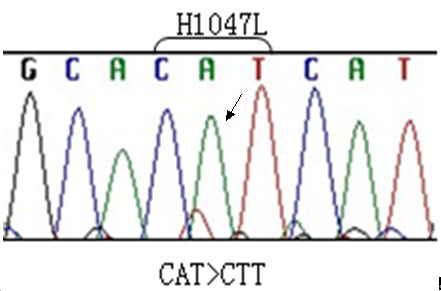


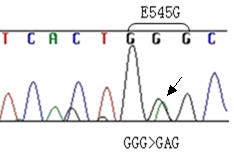


Figure 3. Sequencing results for *PIK3CA* mutations
