# Supplementary material for: KRAS, BRAF and PIK3CA Mutations and the Loss of PTEN Expression in Chinese Patients with Colorectal Cancer
Source: PLoS One. 2012 May 7;7(5):e36653. doi: 10.1371/journal.pone.0036653 (PMC3346734; doi:10.1371/journal.pone.0036653)
Supplement: Appendix S3 — Immunohistochemical staining of PTEN in colorectal cancer tissue and normal tissue. (DOC) [file pone.0036653.s003.doc]

| Negative  (-) | 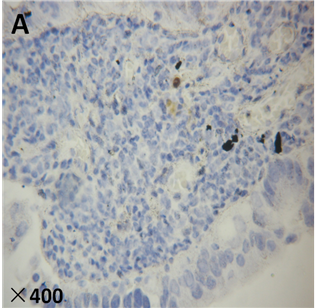 | 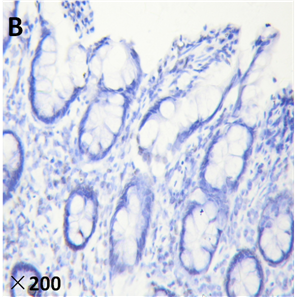 |
| --- | --- | --- |
| Weak  (+) | 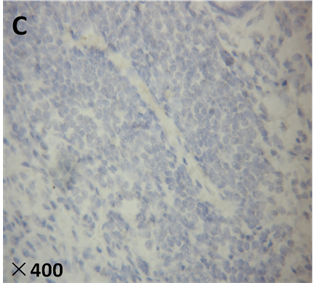 | 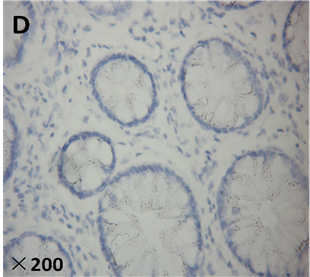 |
| moderate (++) | 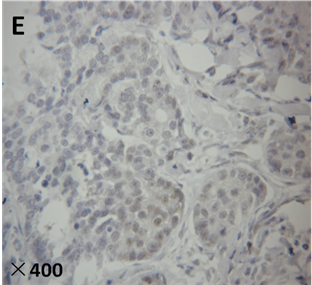 | 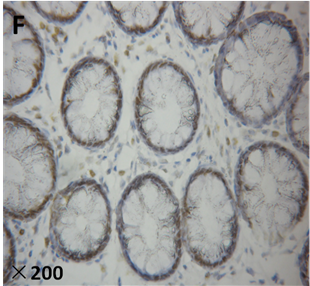 |
| strong (+++) | 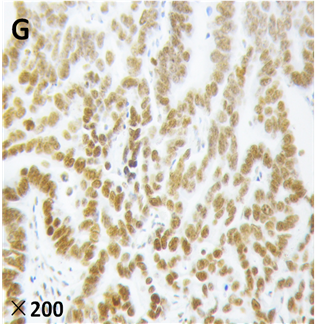 | 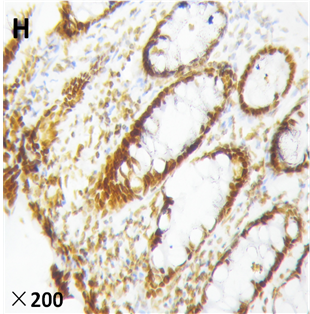 |
| Figure 1 Immunohistochemical staining of PTEN in colorectal cancer tissue and normal tissue; A, C, E, G are cancer tissues and B, D, F, H are normal tissues; A, B with negative PTEN expression (-); C, D with weak PTEN expression (+); E, F with moderate PTEN expression (++); G, H with strong PTEN expression (+++). | | |
